# Micronuclei in Exfoliated Buccal Cells of Children Living in a Cluster Area of Salento (Southern Italy) with a High Incidence of Lung Cancer: The IMP.AIR Study

**DOI:** 10.3390/ijerph15081659

**Published:** 2018-08-05

**Authors:** Adele Idolo, Tiziana Grassi, Francesco Bagordo, Alessandra Panico, Mattia De Giorgi, Francesca Serio, Marcello Guido, Prisco Piscitelli, Giovanni De Filippis, Annamaria Raho, Antonella De Donno

**Affiliations:** 1 Department of Biological and Environmental Science and Technology, University of Salento, via Monteroni 165, 73100 Lecce, Italy; adele.idolo@unisalento.it (A.I.); tiziana.grassi@unisalento.it (T.G.); alessandra.panico@unisalento.it (A.P.); mattia.degiorgi@unisalento.it (M.D.G.); francesca.serio@unisalento.it (F.S.); marcello.guido@unisalento.it (M.G.); antonella.dedonno@unisalento.it (A.D.D.); 2 Interuniversity Research Centre on Influenza and other Tnsmitted Infections (CIRI-IT), 16132 Genoa, Italy; 3 Local Health Authority ASL LE, 73100 Lecce, Italy; priscofreedom@hotmail.com (P.P.); giov.defilippis@gmail.com (G.D.F.); annamaria.raho@gmail.com (A.R.); 4 Euro Mediterranean Scientific Biomedical Institute, 72100 Brindisi, Italy

**Keywords:** micronucleus, exfoliated buccal cells, children, Salento, IMP.AIR study

## Abstract

During the years 2014–2016 the University of Salento performed the “Impact of Air Quality on Health of Residents in the Municipalities of Cutrofiano, Galatina, Sogliano Cavour, Soleto and Sternatia” (IMP.AIR) study, an epidemiological-molecular research project aiming to evaluate early DNA damage in children living in an area of Salento with high incidence of lung cancer among the male population. One hundred and twenty-two children aged 6–8 years attending primary school were enrolled and the frequency of micronucleated cells (MNC) in oral mucosa was evaluated. In addition, a questionnaire was administered to parents to obtain information about personal data, anthropometric characteristics and lifestyles (physical activity, food habits, family context) of the children and perform a multivariate analysis to detect any factors associated with MNC occurrence. Data on airborne pollutants detected in the study area were acquired by the Regional Agency for the Environmental Protection. The presence of MNC was highlighted in about 42% of children with a mean MNC frequency of 0.49‰. The frequency of MNC was associated to obesity, consumption of red or processed meat and having a mother who smokes. Moreover, the prevalence of biomarkers was higher than in another area of Salento not included in the cluster area.

## 1. Introduction

Cancer constitutes an enormous burden on society in more and less economically developed countries alike. The occurrence of cancer is increasing because of the growth and aging of the population, as well as an increasing prevalence of epigenetic factors including those associated with urbanization and economic development [1].

Several decades ago lung cancer emerged as the most common cancer worldwide. In 2012 it was the most common cancer both in term of new cases (1.8 million cases, 12.9% of total) and deaths (1.6 million deaths, 19.4%) [2]. Time trends in lung cancer incidence and mortality vary substantially for different areas and often depends on socio-economic conditions, behavioral factors widespread in the population and environmental conditions. Known risk factors for lung cancer include tobacco smoking [3], exposure to occupational and environmental carcinogens such as asbestos, arsenic, radon, and polycyclic aromatic hydrocarbons [4], indoor air pollution from unventilated coal-fueled stoves and cooking fumes [5,6]. Recently, outdoor pollution and particulate matter have also been determined to cause lung cancer [7]. 

Children are a high-risk group with regards to the short-term and long-term health effects of exposure to toxic pollutants [8,9,10] Indeed, some studies suggest that early exposure during childhood can play an important role in the development of chronic diseases in adulthood: the earlier the exposure, the greater the risk of chronic disease, including cancer [11]. 

For the prevention of cancer it is important to identify any risk conditions early in order to intervene on the modifiable factors responsible for the disease. Molecular epidemiology could represent an innovative and effective approach for primary prevention which may overcome the limits of classical epidemiology based on health damages that have already occurred. In fact, it allows for the evaluation of early molecular alterations predictive of the onset of tumor pathologies [12].

A tumor is the outcome of a degenerative process involving successive generations of cells, which are progressively further advanced towards cancerous growth. It follows multiple exposures to risk factors which initially produce alterations and/or DNA damages in target cells or tissues. The assessment of these biological alterations by means of biomarkers would allow for early detection of health risk condition before they could result in irreversible damage [13]. 

Among biomarkers of early biological effect, the buccal micronucleus cytome (BMCyt) assay [14] has been widely used in studies for measuring environmental genotoxicity [15,16]. Micronuclei appear in the cytoplasm of interphasic cells as additional nuclei, smaller than main nucleus. They are formed of acentric chromosomal fragments or whole chromosomes that are not included in the main daughter nuclei during nuclear division and reflect clastogenic and/or aneugenic events. Micronucleated cells (MNC) can be investigated in target tissues, such as oral mucosa, especially exfoliated buccal cells (EBC) in which they highlight the presence of alterations in chromosomal structure and oxidative stress caused by exposure to air pollutants [17,18,19]. The frequency of MNC could be increased also by altered health states, such as obesity [20] and some diseases or treatments [21], and unhealthy lifestyles, such as residence in areas with high traffic levels [19], smoking and exposure to passive smoking [22,23], cooking fumes [24] and heat-processed foods [25]. In contrast, a high level of parental education [26], a healthy diet [27] and physical exercise [23] may contribute to decrease the occurrence of MNC. 

Salento is a geographical region located in the southern part of Apulia (south-eastern Italy) including the provinces of Taranto, Brindisi and Lecce. Although the Province of Lecce is characterized by low industrial activity, the mortality rates for lung cancer were found to be significantly higher than regional and national rates [28]. In particular, a geographical cluster including sixteen municipalities with a high incidence of lung cancer (355 confirmed cases vs. 285 expected in the period 2003–2006) and other respiratory diseases in the male population has been recently identified [29]. 

This paper reports the results of the “Impact of Air Quality on Health of Residents in the Municipalities of Cutrofiano, Galatina, Sogliano Cavour, Soleto and Sternatia” (IMP.AIR) study an epidemiological molecular research performed in the years 2014–2016 by the University of Salento and aimed to: (i) evaluate the frequency of MNC in oral mucosa of 6–8-year-old children living in the five Municipalities included in the cluster area and (ii) assess lifestyles of the children that could have effects on the measured biological responses. The results were then compared with those found in children living in an area of Salento not included in the cluster area and sampled simultaneously by the same research group as part of another study (the MAPEC_LIFE study) [12].

## 2. Materials and Methods 

### 2.1. Study Design

**Figure 1:**
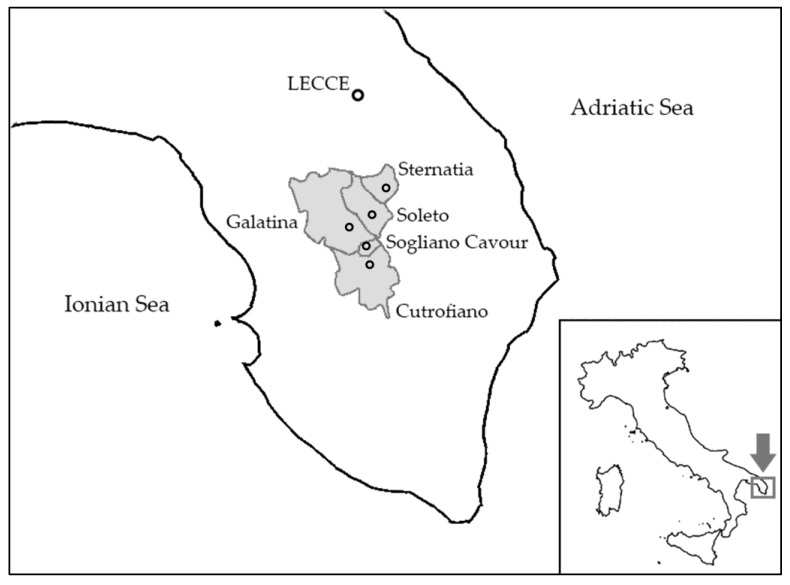
Map of the Salento Peninsula showing the location of the municipalities included in the IMP.AIR study and their administrative area (in grey).

A group of children aged 6–8 years living in the Municipalities of Cutrofiano, Galatina, Sogliano Cavour, Soleto and Sternatia was enrolled and the early DNA damage in their EBC was evaluated. Individual characteristics and lifestyles of children as well as information on air quality in the area were also acquired. These municipalities are less than 10 km from each other and together have a population of 48,084 inhabitants, 60% of which concentrated in the town of Galatina (Figure 1). The territory includes several urbanized areas and an industrial site with cement factories and plants that use waste fuel that affect microbial and chemical quality of the environment [30,31]. The data contained in the mortality tables of the Regional Epidemiological Observatory (OER-Puglia) [32] show that, overall, in the five municipalities involved in the IMP.AIR study, the number of lung cancer deaths observed in the male population in the decade 2000–2009 was 284, significantly higher (*p* < 0.01) than expected deaths (197.7), with a standardized mortality ratio (SMR) of 143.5.

Finally, a comparison between the results concerning the early DNA damage in children recruited in the IMP.AIR study and those detected in children recruited in an area of Salento not included in the cluster area [33] was performed. The reference cohort was the one recruited in the MAPEC_LIFE study [34], which includes 213 children aged 6–8 years living in Lecce. The EBC of these children were sampled in May 2105 and submitted to the BMCyt assay by the same research group as the IMP.AIR study. Lecce is a city about 20 km away from the study area with a population of about 90,000 inhabitants. Data concerning the air quality detected by the Regional Agency for Environmental Protection (ARPA Puglia) in this town [35] highlight that in 2015 the level of pollutants was always lower than the limits set by European Directive 2008/50/EC [36]. In addition, lung cancer mortality among the male population in the years 2000–2009 was significantly lower (*p* < 0.01) than in the study area with a SMR of 114.6 [32].

### 2.2. Recruitment

The study was performed on children that in the school year 2014–2015 attended the first, second and third classes of the primary schools in Cutrofiano, Galatina, Sogliano Cavour, Soleto and Sternatia. The study population was part of the 6–8-years-old student body counting 1213 children, 622 males (51.3%) and 591 females (48.7%). In the five municipalities involved in the study, six primary schools were selected: two schools in Galatina and one in each other municipality.

The parents of all the children attending the first three classes of these schools were informed about the study and invited to participate asking them to sign the Consent Form, after verifying that their children did not fall into exclusion criteria (age below 6 years or equal/above 9, residence in cities other than those involved in the study, the presence of serious illness, exposure to radiotherapy or chemotherapy in the 12 months preceding the investigation, exposure to radiographic testing in the month preceding the investigation, use of dental braces).

### 2.3. Questionnaire Administration

The parents who agreed to the participation of their children in the study were asked to fill in a validated questionnaire [37]. The questionnaire contained 148 questions, subdivided into various sections: child’s personal information (gender, date and nation of birth, height and weight); child’s health status; domestic environment (intensity of traffic near the home, including heavy goods vehicles, fuel used for heating and cooking, use of fireplaces, presence of gas boilers inside the dwelling, presence of smokers inside the dwelling, use of solvents for hobbies, i.e. paint thinner, white spirit, acetone, turpentine, toluene, etc.); information on the child’s lifestyle (sports, outdoor exercise, staying in the kitchen during food cooking); parents’ characteristics (nation of birth, level of education, occupation, smoking habits); children’s dietary habits with particular reference to consumption of dishes cooked by methods considered “risky” for the production of toxic substances (i.e., barbecued, griddled, fried, toasted or smoked) [25]. Each questionnaire was assigned an alphanumeric code that was also used to identify the biological sample associated with the same child.

### 2.4. Collection of Biological Samples

Children whose parents correctly completed the questionnaire were invited to provide biological samples in May 2015. They were asked to rinse the mouth twice with mineral water. A small-headed toothbrush was used to collect epithelial buccal cells, by gently scraping (10 times in a circular motion), from the inner surface of both cheeks. The head of the brush was then dipped into tubes containing 15 mL of Saccomanno’s fixative (50% ethanol, 2% polyethylene glycol, *v*/*v*; solution diluted in water and stored at 4 °C) and rotated repeatedly to dislodge and release the cells into the buffer. The samples were stored at 4 °C and subjected to biological assay in 7 days.

### 2.5. Buccal Micronucleus Cytome (BMCyt) Assay

The BMCyt assay was performed according to the procedure described by Thomas and Fenech [14]. The cell suspensions were centrifuged, washed twice with PBS (Invitrogen Srl, Milan, Italy), filtered through a 100 µm nylon filter (Merck Spa, Milan, Italy) and centrifuged again. Cell pellets were resuspended in ice-cold PBS. Cell count was then performed using a hemocytometer (Burker chamber) to verify the cell concentration. Buccal cells were then fixed with ice-cold Carnoy’s fixative (methanol and glacial acetic acid 3:1). For each sample, two slides were prepared by smearing 100 μL of cell suspension (approximately 10^5^ cells/slide). The slides were treated with Schiff’s reagent (Sigma-Aldrich, Milan, Italy), washed, stained with 0.2% Light Green reagent (Sigma-Aldrich), air dried and finally mounted with DePex mounting medium (VWR International PBI Srl, Milan, Italy).

**Figure 2:**
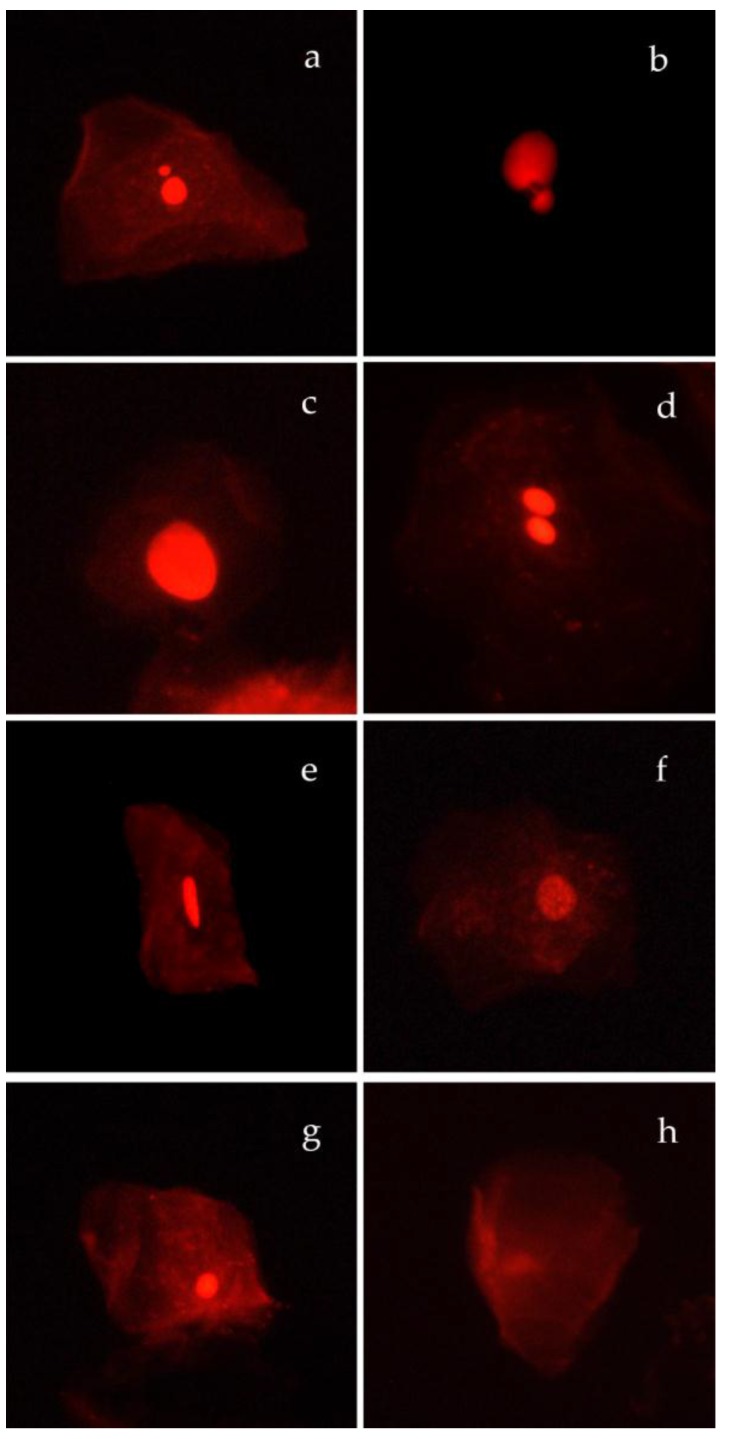
Cell types classified according to Bolognesi et al. [38]: (**a**) Cell with a micronucleus (MNC); (**b**) cell with a nuclear bud (NBUD); (**c**) basal cell (BC), (**d**) binucleated cell; (BNC); (**e**) condensed chromatin cell (CCC); (**f**) karyorrhectic (KHC); (**g**) pyknotic (PYK); (**h**) karyolytic (KYL).

Cells were scored using both bright-field and fluorescence microscopy (Eclipse 50i, Nikon, Tokyo, Japan) according to Thomas and Fenech [14]. Cell types characterized by anomalies associated with cell death and nuclear abnormalities indicative of chromosomal instability or DNA damage were classified according to Bolognesi et al. [38]. Micronucleated cells (MNC) and nuclear buds (NBUD) as well as basal cells (BC), binucleated cells (BNC), condensed chromatin cells (CCC), karyorrhectic (KHC), pyknotic (PYK) and karyolytic (KYL) (Figure 2) were scored and their frequency was reported as number of classified cells in 1000 differentiated cells (‰).

### 2.6. Air Quality

Information about air quality in the study area was obtained by acquiring hourly data on the air pollutants registered by the permanent monitoring station “I.T.C. La Porta” located in Galatina and belonging to the air quality monitoring network of the Regional Agency for the Environmental Protection (ARPA Puglia). Following parameters were taken into account: fine particulate matter (PM_2.5_), nitrogen dioxide (NO_2_), carbon monoxide (CO), sulphur dioxide (SO_2_) and ozone (O_3_). For PM_2.5_, NO_2_, CO and NO_2_, 24-h mean and annual mean were calculated. For O_3_ hourly running eight-hour averages were calculated and the maximum daily eight-hour mean was taken into account. Finally, for each parameter the compliance with the limit (target value) established by European Directive 2008/50/EC [36] was verified.

### 2.7. Data Analysis

All the results obtained from the biological survey and administered questionnaires were entered into a Microsoft Excel database and statistically processed using MedCalc Software version 12.3 (MedCalc Software BVBA, Ostend, Belgium).

The age of each child was measured by calculating the time interval between the date of birth given on the questionnaire and the date of filling the questionnaire. The data for weight and height given by the parents were used to calculate the children’s body mass index (BMI) (weight (kg)/height (m^2^)). Weight status was identified in accordance with the indications of the International Obesity Task Force (IOTF) that define “overweight” (OW) and “obese” (OB) with reference to the BMI threshold values for boys and girls aged 2–18 years on the basis of adult values (OW = 25 kg/m^2^; OB= 30 kg/m^2^) [39]. The cut-off for the underweight (UW) category was set at the “−2 z-score” on the basis of the BMI threshold values set out in Cole et al. [40].

The different foods listed in the section of the questionnaire related to children dietary habits were divided into the following categories: bread, pasta/rice/soups, pizza and focaccia, legumes, vegetables, potatoes, fresh fruit, dried fruit, red and processed meat, poultry, fish, eggs, milk and dairy products, cakes, snacks, soft/fizzy drinks, condiments.

For each item there were seven different options concerning the frequency of consumption, listed in ascending order from “never” to “2 times a day”. For the olive oil the consumption unit corresponded to 1 tablespoon of oil (about 10 g) while for the butter to a knob of butter (about 10 g). For soft/fizzy drinks two additional consumption categories were considered, “4 times a day” and “6 times or more a day”. The frequency of consumption of each food and beverage category was then transformed as follows: the frequency value “never” was transformed to “0 times per week”, “less than 1 time per week” was transformed to “0.5 times per week”, “1–2 times per week” was transformed to “1.5 times per week”, “3–4 times per week” became “3.5 times per week”, “5–6 times per week” became “5.5 times per week”, “once per day” became “7 times per week” and “2 times per day” became “14 times per week”, “4 times per day” was transformed to “28 times per week” and “6 times per day” was transformed to “42 times per week”.

The answers in the questionnaires and the results of biological analyses were analyzed in order to assess the average values and the standard deviation (SD) of quantitative variables (i.e., age, height, weight, BMI, frequency of cell types), and the relative frequency (%) of qualitative variables. 

One-way ANOVA was performed to verify any differences between results of BMCyt in IMP.AIR study and in MAPEC_LIFE study. Multivariate logistic regression analyses with the corresponding odds ratio (OR) and a 95% confidence interval (CI) were carried out to examine the possible association between personal, socioeconomic, behavioral, and environmental factors (independent variables) and positivity for the BMCyt assay (dependent variable).

### 2.8. Ethical Aspects

The study was approved by the Ethical Committee of the Lecce Local Health Authority (ASL) with report no. 68 of 27/02/2013. Participation in this study was voluntary. All the parents of the selected classes received the information of the study and, in the case of acceptance of the participation, expressed their consent to the processing of the personal data by signing the relevant form. All the data were collected and analyzed confidentially, in accordance with Italian Legislation on protection of personal data, for research purposes.

## 3. Results

### 3.1. Characteristics of Recruited Children

In total, 122 children (10% of the student population aged 6–8 years), 121 born in Italy (99.2%), participated in the study. Of these, 67 (54.9%) were male and 55 (45.1%) females. At the time of recruitment, 34 children (27.9%) were 6 years old, 41 (33.6%) were 7 years old and 47 (38.5%) were 8 years old with an average age of about 7.65 years (Table 1).

The recruited children had an average weight of 28.3 ± 7.6 kg, an average height of 127.2 ± 8.7 cm and an average BMI of 17.247 ± 2.892 kg/m^2^. 63.1% of them were normal weight, 32.0% were in excess weight, included obese (14.8%), while 4.9% were underweight.

Sixty-five children (53.3%) performed regular sports at least 3 times a week; of these, 38 (31.7%) practiced outdoor sports and 11 (9.0%) swimming. Out of the entire group of children, 69.7% played outdoor every day for more than one hour.

### 3.2. Dietary Habits

The average weekly consumption ± standard deviation (SD) of foods and beverages by the children participating in the study is shown in Table 2. 

On average, fresh fruit, milk and diary products, cakes, pasta/rice/soups were consumed more than once a day; bread, red or processed meat about once a day; soft or fizzy drinks about 5 times a week; pizza and focaccia, vegetables, potatoes, dried fruit, poultry, fish, eggs, snacks one to four times a week; legumes less than once a week. As for the condiments, olive oil (7.97 tablespoons of oil per week) was used more frequently than butter (1.05 knobs of butter per week).

In the month preceding the survey many children consumed foods prepared by “risky” cooking methods (Table 3). In particular, 91% of children consumed fried foods, 79.5% wood-fired pizza and 59.0% grilled foods.

### 3.3. Domestic Environment

Data relating to the domestic environment (Table 4) show that 27.9% of the children lived in areas perceived by their parents as high-traffic areas and 15.7% near roads with a high density of heavy vehicles. The fuel used for heating the house was mainly methane gas (82.3%), followed by wood (9.2%), diesel (3.8%) and electricity (3.8%), while fuel used for cooking was almost exclusively methane gas (91.0%). The fireplace was present in 61 (50.0%) homes where it was used on average 4.1 days in the month preceding the survey (not in the table). Out of all children in the study, 3.3% lived in houses equipped with a gas boiler, 20.5% lived with at least one person who smoked inside the house and 19.2% usually stayed in the kitchen during food cooking. Cooking on griddle or barbecue was done in 68.6% of homes while solvents for hobbies were used in 1.6%.

### 3.4. Characteristics of Parents

Over 90% of the parents were born in Italy (Table 5). Eighty-six mothers (70.5%) and seventy-two fathers (59.5%) had a high school diploma or a higher education certificate, but only 47.5% of mothers worked, compared to 91.4% of the fathers. Smoking habits were more common in fathers (38.8%) than in mothers (12.3%).

### 3.5. Air Quality

Data on environmental pollutants detected by the monitoring station located in Galatina are shown in Table 6. PM_2.5_, NO_2_, CO and SO_2_ have not shown any noncompliance with the limits indicated in the European Directive 2008/50/EC on air quality [36]. On the contrary, maximum daily eight-hour mean value of O_3_ exceeded for 67 times the limit of 120 µg/m³ that should not to be exceeded on more than 25 times per calendar year.

### 3.6. The Buccal Micronucleus Cytome (BMCyt) Assay

Of the 122 samples collected from oral mucosa of the recruited children and submitted to the BMCyt assay, 28 (23.0%) were not suitable for cell counting as the number of cells was insufficient. The frequency of chromosomal and DNA damage markers (MNC, and NBUD), cell proliferation markers (BC and BNC) and cell death/apoptosis markers (CCC, KHC, PYK, and KYL) in 94 scored samples is reported in Table 7. 

Results from analysis performed on BMC of 213 children living in Lecce and included in the cohort of MAPEC_LIFE study [33] were also reported. In the IMP.AIR study MNC were found in 39 (41.5%) samples, NBUDs in 8 (8.5%) samples, BC in 66 (70.2%) samples, BNC in 88 (93.6%) samples, KHC in 91 (96.8%) samples and PYK in 37 (39.4%) samples. CCC and KYL were identified in all the samples (not in table). Overall, the mean MNC frequency was 0.49 ± 0.65‰ with a maximum of 3 MNC‰ in a sample. In the MAPEC_LIFE study the frequency of MNC in 213 children living in Lecce was 0.24 ± 0.32‰, significantly lower (*p* < 0.01) than in IMP.AIR study.

The results of multiple logistic regression (Table 8) revealed that obesity (OR = 3.849; CI = 1.140–12.994), red or processed meat consumption more than 4 times a week (OR = 3.622; CI = 1.154–11.365) and having a mother who smokes (OR = 5.511; CI =1.156–26.274) were strongly associated to MNC occurrence. In contrast, male gender, residence in high traffic areas, use of fireplace more than 8 days a month, vegetables consumption more than 3 times a week, sport activity more than 3 times a week, having a father or a mother with high school diploma and a father who smokes seemed to have no relationship with intra-group variability of MNC frequency.

## 4. Discussion

The IMP.AIR study allowed to evaluate the frequency of markers of chromosomal and DNA damage, cell proliferation, cell death/apoptosis in EBC of 94 of children living in five municipalities (Cutrofiano, Galatina, Sogliano Cavour, Soleto and Sternatia) included in a cluster area of Salento with high incidence of lung cancer. The results showed a mean MNC frequency of 0.49 ± 0.65‰ with a proportion of children with at least one micronucleus in EBC of 41.5%.

These results could be compared with those obtained from a similar study (the MAPEC_LIFE study [12]), conducted in the same period (May 2015), with the same methodology and by the same research group as the IMP.AIR study, on 213 children aged 6–8 years living in Lecce, city not included in the cluster area. In this study, the frequency of MNC was significantly lower (0.24 ± 0.32 MNC‰) [33], suggesting that children living in the cluster area are more likely to have early DNA damage than children living outside of it. As previously stated [16,18,19,42], this may imply an increased risk to develop cancer in adulthood.

The different frequency of MNC may be due to different environmental exposures or lifestyles. Other studies evaluated the frequency of MNC in EBC of school-age children exposed to air pollutants. A higher MNC frequency, in respect of our study, was observed in children with mean age of 7.3 years living close to major freeways and arterial roads in Oakland, California (0.67 ± 1.44 MNC‰) [43] and in 9 years old children living near chipboard industries in the manufacturing district of Viadana, Italy (0.12 ± 0.09 MNC%) [44]. Moreover, a group of Brazilian children aged ≤7 years living in an urban polluted area showed a mean MNC frequency of 1.20 ± 0.83 MNC‰ while those living in a rural nonpolluted area measured 0.19 ± 0.31 MNC‰ [45]. Finally, the level of MNC detected in the IMP.AIR study appeared consistent with that reported during the winter in some Italian cities with high urban air pollution such as Torino (0.39 ± 0.48 MNC‰) and Brescia (0.53 ± 0.61 MNC‰) [33]. In summary, from an environmental point of view, the frequency of biomarkers found in this study appeared to be non-basic and similar to the level recorded in some polluted areas. 

Unfortunately, the information regarding the air quality is poor both quantitatively and qualitatively because in the study area there was only one ARPA monitoring station, which detected only some of the most common atmospheric pollutants. In particular, it was not possible to verify the concentration levels of many pollutants which are necessary to assess the risk for human health associated with exposure to atmospheric pollution such as PM_10_, benzene, polycyclic aromatic hydrocarbons and heavy metals. Moreover, in 2015 the concentrations of PM_2.5_, NO_2_, CO and SO_2_ appeared to be overall acceptable, if compared with limits indicated in the European legislation. On the contrary, ozone often exceeded the target value set in the legislation. Ozone is a secondary pollutant that is formed in the atmosphere by photochemical reactions among other substances (including nitrogen oxides and volatile organic compounds) [46]. The formation process is catalyzed by solar radiation, so the highest concentrations are recorded in the warmer months. High levels of ozone are responsible for acute effects in exposed people and represent an index of possible presence of other pollutants [47].

Many of the children participating in the study were found exposed to unhealthy factors concerning domestic environment or lifestyles. These may have contributed to the occurrence of DNA alterations in EBC of children detectable through MNC.

Multivariate analysis allowed to establish which variables were responsible for intra-group variability of MNC frequency. Assuming that all children were exposed to the same outdoor air pollutants, as they all lived in a small geographic area, the occurrence of MNC appeared to be related to obesity, consumption of red or processed meat and exposure to the smoke of the mothers. 

In the literature a positive correlation between weight excess and MNC frequency was described in oral mucosa cells [20] of school-aged children. In our study 35% of the children were overweight and even 24% were obese. These percentages were higher than the national averages but in line with studies showing a high frequency of overweight children in the southern regions [48,49]. 

The intake of red or processed meat, especially when it was more than 4 servings a week, was also demonstrated to be related to MNC occurrence. The role of the consumption of this foods in colorectal cancer development was previously highlighted as well as their relationship with early genotoxic effects such MNC formation [50]. In addition, most children showed other unhealthy eating habits such as (i) frequent consumption of foods subject to cooking methods considered “risky” (barbecued, griddled, fried, toasted, smoked) for the production of genotoxic compounds such as acrylamide, heterocyclic amines, nitrosamines and polyaromatic hydrocarbons [25]; (ii) high intake of cakes and soft/fizzy drinks and low intake of legumes that, together with high intake of red meat, were far from the principles of the Mediterranean diet, a dietary model able to counteract DNA damage including MNC formation [27] but frequently disregarded by Italian children [51].

Many children participating in the study were exposed to environmental tobacco smoke as they lived together with at least one smoker. While in public places the exposure to passive smoking has been strongly limited by legislation, private homes still remain a site where children could be dangerously exposed to environmental tobacco smoke [52]. This includes the so called “second-hand smoke” (mixture of the smoke from the burning tip of a cigarette and the smoke exhaled by a smoker) [41] and “third-hand smoke” (tobacco smoke pollutants remaining on surfaces, including skin and clothes, and in dust in an indoor environment) [53,54]. In our study, smoking parents (about 40% of fathers and 12% of mothers) were more numerous than in other studies conducted on Italian children of the same age [34]. As previously described [22], exposure to second- or third-hand smoke could influence the frequency of MNC in children’s oral mucosa cells. However, in this study only the exposure to mother smoke was strictly related to MNC occurrence. The association between smoking mothers and genotoxic damage in children was already highlighted [23] and was probably due to the closer relationship between mothers and young children, both for the longer time spent together and because mothers often carry out certain tasks more than fathers such as food preparation or hygiene of children.

No other individual or behavioral factors (such as gender, traffic levels, physical activity, fuel used for heating the house, socio-cultural level of the parents and other lifestyles) were found associated with MNC occurrence. In particular, as previously found in EBC of school-age children [42], we observed in male subjects a number of chromosomal alterations not substantially different than in females. Therefore, among children aged 6–8 years living in the cluster area there were no differences in the frequency of biomarkers that could justify the epidemiological differences related to lung cancer between the male and female population.

## 5. Conclusions

The results of the IMP.AIR study showed that children aged 6–8 years living in an area of Salento with a high incidence of lung cancer among the male population had a higher frequency of MNC than children living in other areas of Salento not included in the cluster area or in non-polluted areas. Some individual or poor lifestyle factors, such as obesity, high consumption of red or processed meat and exposure to environmental tobacco smoke, may have contributed to the increased frequency of MNC. However, further and appropriate investigations are necessary in order to define in more detail the cause-effect relationship between environmental/individual factors and the health outcomes. In particular, it would be advisable to intensify the routine monitoring of airborne pollutants both quantitatively (number of monitoring stations) and qualitatively (substances to be researched), also considering the epidemiology of chronic respiratory diseases recorded in this area. Moreover, since MNC have proved to be a good indicator of genotoxic damage, it would be suitable to continue the epidemiological-molecular surveillance, extending it to other areas of Salento.

In any case, there is a need to perform communication and training programs for various targets (children, parents, teachers) on the prevention of risks to human health associated with air pollution and incorrect lifestyles which have previously proved to be useful for improving knowledge on these topics [55,56,57].

## Figures and Tables

**Table 1:** Data on the personal and behavioral variables of the children participating in the study.

Variable	Units of Measure	Value
Males	N (%)	67 (54.9%)
Females	N (%)	55 (45.1%)
Born in Italy	N (%)	121 (99.2%)
6 years old	N (%)	34 (27.9%)
7 years old	N (%)	41 (33.6%)
8 years old	N (%)	47 (38.5%)
Average age	years ± standard deviation (SD)	7.65 ± 0.94
Average weight	kg ± SD	28.3 ± 7.6
Average height	cm ± SD	127.2 ± 8.7
Average BMI	kg/m^2^ ± SD	17.247 ± 2.892
UW	N (%)	6 (4.9%)
NW	N (%)	77 (63.1%)
OW	N (%)	21 (17.2%)
OB	N (%)	18 (14.8%)
Sports (≥3 times/week)	N (%)	65 (53.3%)
Outdoor sports	N (%)	38 (31.7%)
Swimming	N (%)	11 (9.0%)
Outdoor play (>1 h/day)	N (%)	85 (69.7%)

**Table 2:** Average weekly consumption of foods and beverages by the recruited children.

Food Categories	Average Consumption ± SD (times/week)
Bread	6.97 ± 3.79
Pasta/rice/soups	10.36 ± 4.66
Pizza and focaccia	1.70 ± 0.91
Legumes	0.80 ± 0.73
Vegetables	9.55 ± 7.88
Potatoes	1.51 ± 1.30
Fresh fruit	20.67 ± 23.08
Dried fruit	1.10 ± 1.56
Red or processed meat	6.48 ± 3.62
Poultry	1.83 ± 1.19
Fish	3.74 ± 2.67
Eggs	1.33 ± 0.68
Milk and dairy products	16.42 ± 9.05
Cakes	14.28 ± 9.37
Snacks	1.88 ± 1.69
Soft/fizzy drinks	5.13 ± 6.64
Olive oil ^1^	7.97 ± 4.31
Butter ^2^	1.05 ± 1.85

^1^ Tablespoons (about 10 g) of olive oil/week. ^2^ Knobs (about 10 g) of butter/week.

**Table 3:** Children who consumed foods subject to risky cooking methods in the month before the survey.

Variable	*N* (%)
Fried foods	111 (91.0)
Wood-fired pizza	97 (79.5)
Barbecued foods (wood/charcoal)	72 (59.0)
Foods cooked on the griddle	59 (48.4)
Toasted bread	45 (36.9)
Smoked foods	23 (18.9)

**Table 4:** Prevalence of exposure factors linked to the home context among the children participating in the IMP.AIR study.

Variable	*N* (%)
Residence in high-traffic areas	34 (27.9%)
Residence near roads with high traffic of heavy vehicles	19 (15.7%)
Fuel used for heating	
Methane gas	107 (82.3%)
Wood	12 (9.2%)
Diesel	5 (3.8%)
Electricity	5 (3.8%)
Fuel used for cooking	
Methane gas	121 (91.0%)
Electricity	9 (6.8%)
Wood	3 (2.3%)
Presence of fireplace	61 (50.0%)
Presence of gas boiler inside home	4 (3.3%)
Living with smokers inside the dwelling	25 (20.5%)
Frequent staying in the kitchen during food cooking	23 (19.2%)
Cooking on the griddle/barbecue	83 (68.6%)
Use of solvents for hobbies	2 (1.6%)

**Table 5:** Prevalence of variables regarding parents of children included in the IMP.AIR study.

Variable	Mother *N* (%)	Father *N* (%)
Born in Italy	110 (90.2%)	112 (92.6%)
High school education or greater	86 (70.5%)	72 (59.5%)
Employment rate	58 (47.5%)	106 (91.4%)
Smoking habits	15 (12.3%)	47 (38.8%)

**Table 6:** Data on environmental pollutants detected by the monitoring station located in Galatina.

Variable	PM_2.5_ (µg/m³)	NO_2_ (µg/m³)	CO (mg/m³)	SO_2_ (µg/m³)	O_3_ * (µg/m³)
Annual mean	17.2	11.4	0.51	4.23	103.3
Maximum 24-h mean value	60.2	29.3	1.3	8.48	166

* Maximum daily eight-hour mean.

**Table 7:** Frequency ± SD of chromosome damage markers (MNC and NBUD), cell proliferation markers (BC and BNC), and cell death markers (CCC, KHC, PYK and KYL) in exfoliated buccal cells (EBC) of children involved in the IMP.AIR study compared with children living in Lecce and included in the cohort of MAPEC_LIFE study [41].

Biomarkers (‰) ^1^	IMP.AIR Study ^2^	Lecce (MAPEC_LIFE Study) ^3^ [33]
MNC	0.49 ± 0.65	0.24 ± 0.32 *
NBUD	0.10 ± 0.33	0.09 ± 0.26
BC	1.70 ± 1.72	0.19 ± 0.41 *
BNC	2.73 ± 1.79	3.26 ± 1.88
CCC	51.20 ± 17.97	26.60 ± 18.59 *
KHC	8.50 ± 5.74	13.29 ± 13.90 *
PYK	0.64 ± 1.00	0.11 ± 0.31 *
KYL	16.00 ± 4.76	28.62 ± 21.05 *

^1^ MNC, micronuclei; NBUD, nuclear buds; BC, basal cells; BNC, binucleated cells; CCC, condensed chromatin cells; KHC, karyorrhectic cells; PYK, pyknotic cells; KYL, karyolytic cells. ^2^ Sampled children (n) = 94. ^3^ Sampled children (n) = 213. * *p* < 0.01 (one-way ANOVA)

**Table 8:** Multivariate logistic regression analyses, with the corresponding odds ratio (OR) and a 95% confidence interval (CI), between personal or behavioral factors (independent variables) and positivity for the BMCyt assay (dependent variable).

Independent Variables	OR	CI (95%)	*p*-Value
Male	0.802	0.293–0.195	0.667
Obesity	3.849	1.140–12.994	0.030
Residence in high traffic areas	1.624	0.539–4.897	0.389
Use of fireplace (>8 days/month)	3.585	0.724–17.756	0.118
Red or processed meat consumption (>4 times/week)	3.622	1.154–11.365	0.027
Vegetables consumption (>3 times/week)	0.378	0.122–1.175	0.093
Sport (≥3 times/week)	0.692	0.241–1.982	0.492
Father with high school diploma	0.873	0.308–2.478	0.799
Mother with high school diploma	1.672	0.462–6.043	0.433
Smoking father	0.955	0.346–2.637	0.929
Smoking mother	5.511	1.156–26.274	0.032
